# M. C. McNamara, D.D.S.

**Published:** 1901-10

**Authors:** 


### M. C. McNAMARA, D.D.S.

Dr. M. C. McNamara, an old resident of St. Louis, died June 16, 1901, at Alexian Brother's Hospital, of paralysis. In his prime he was one of the most successful dentists, having at one time an income of not less than twenty thousand dollars a year. The St. Louis Dental Society held a special meeting at Dr. Conrad's office, 3666 Olive Street, on the 17th. After several short addresses by a number of the members, the following were chosen to act as pall-bearers: O. H. Manhard, William Conrad, Walter M. Bartlett, John H. Kennerly, B. L. Thorpe, P. H. Eisloffel, John G. Harper, Abel J. Prosser, and A. Tschirner. A committee was appointed to draft a biographical sketch of the deceased. The following is their report:

"Dr. M. C. McNamara was born in 1829, in Ontario, Canada, of Irish parentage. He engaged in mercantile pursuits there in his earlier years, and there married Miss Katherine Aquesta Martin, fourth daughter of William Francis Martin, a civil engineer in the employ of the British government. He was also a member of the Council in London, Canada.

"Dr. McNamara in 1863 removed to Philadelphia, and a year later came to St. Louis. Soon after his arrival here Dr. McNamara was graduated from the old St. Louis Dental College, and began the practice of dentistry. Later he occupied a chair in the College.

"In 1896 Dr. McNamara lost his wife, and since then has taken little part in active business. He was a member of the Knights of St. Patrick, and of a number of charitable and Catholic religious organizations. He was a former president of the St. Louis Dental Society, a member of the Odontological Society of St. Louis, and of the Missouri State Dental Association.

"Doctor McNamara had a family of seven sons and two daughters. The survivors are four sons—Joseph T., John J., William F., and Edward J.—and two daughters,—Miss Frances K. McNamara and Mrs. F. L. Linton.

"Dr. McNamara was an all-round dentist, and took great pride in making his operations as near perfect as possible. He was ethical in every sense of the word. For a number of years he insisted on entertaining the St. Louis Dental Society at its annual meetings for the election of officers. These meetings were held at his spacious residence; after the business of the session was disposed of an elegant spread was partaken of in the dining-room.

"Personally Dr. McNamara was a fine specimen of an Irish gentleman and generous to a fault.

"Having been long connected with the Jesuit Church at Ninth and Washington Avenue, by special permit he was buried from St. Francis Xavier Church, Grand and Lindell Boulevard, Rev. Father Daniel McErlane, S.J., officiating, and was interred in Calvary Cemetery, Tuesday, June 18, 1901.

"John G. Harper,
"William N. Conrad,
"Adam Flickenger,
*"Committee."*

